# Erythroleukemia: Classification

**DOI:** 10.1002/jha2.676

**Published:** 2023-03-17

**Authors:** Nathalie Cervera, Arnaud Guille, José Adélaïde, Marie‐Anne Hospital, Sylvain Garciaz, Marie‐Joelle Mozziconacci, Norbert Vey, Véronique Gelsi‐Boyer, Daniel Birnbaum

**Affiliations:** ^1^ Laboratoire d'Oncologie Prédictive Centre de Recherche en Cancérologie de Marseille (CRCM) Institut Paoli‐Calmettes INSERM UMR 1068 CNRS UMR725 Aix‐Marseille Université Marseille France; ^2^ Département d'Hématologie Institut Paoli‐Calmettes Marseille France; ^3^ Département de BioPathologie Institut Paoli‐Calmettes Marseille France

**Keywords:** acute erythroid leukemia, molecular classification, mutations, TP53

## Abstract

Acute erythroid leukemia (AEL) is a rare (2%–5%) form of acute myeloid leukemia (AML). Molecular alterations found in AEL resemble those of other AMLs. We report a classification of AELs in three major classes, with different prognosis and some specific features such as a tendency to mutual exclusion of mutations in epigenetic regulators and signaling genes.

1

Acute erythroid leukemia (AEL) is a rare (2%–5%) and particular form of acute myeloid leukemia (AML) due to a predominant bone marrow erythroid proliferation. The definition of AEL has undergone several revisions from the first description in 1976 by the French‐American‐British cooperative group classification till nowadays. Two subtypes were recognized by the 2001 World Health Organization [1]. The first type, acute erythroid/myeloid leukemia (so‐called M6a), is defined by the presence of 50% or more of erythroid precursor and 30% or more of blasts among the nonerythroid component. The second type is pure erythroid leukemia (so‐called M6b) in which 80% or more of all marrow cells are immature erythroid precursors with minimal differentiation and without a myeloblasts increase. In both cases, dysplasia can be observed, especially dyserythropoiesis. M6b are extremely rare and both M6a and M6b share a poor prognosis. Classification of M6a has already been a matter of debate (are they myelodysplastic syndromes [MDS] or AML ?) with an arbitrary calculation of the % of blasts. In the 2016 WHO classification [2], the criteria for the diagnosis of erythroleukemia changed, moving cases previously diagnosed as the erythroid/myeloid type to MDS and with the conservation of M6b recognized as the only subtype of AML not otherwise specified. For the cases with more than 20% of blasts and ≥50% of erythroblast, morphological diagnosis is AML. Recently, the fifth edition of the WHO classification of hematolymphoid tumors [3] retained this change and the definition of pure erythroleukemia, now called acute erythroid leukemia (≥80% of erythroblasts of which ≥30% are proerythroblasts).

Molecular characterization has shown that AELs from adult patients have more or less the same mutations as the other AMLs [4, 5, 6, 7]. In 2016, we proposed a molecular classification of M6a‐AELs into three main classes, *NPM1*‐mutated, secondary‐like, and*TP53*‐mutated, and an additional fourth class for some cases that could not be included [5]. We also suggested that the erythroid phenotype could be due to specific additional mutations such as alterations in the EPOR/JAK2 pathway [8], a different cell‐of‐origin or both [9]. Thus, while AEL pathogenesis remains unclear, it may be linked to cellular pliancy.

We have refined here our three‐class molecular classification of AELs by analyzing a series of 166 samples (including M6a and M6b) from the following series: (1) AELs, we had previously analyzed by targeted next generation sequencing (tNGS) and array‐comparative genome hybridization (aCGH) [5] and five new cases (Table S1) (2) AELs reported in a recent study by Takeda [10] analyzed by whole exome sequencing and/or whole genome sequencing. Informed consent was obtained for all samples. To compare and pool the results on our samples (*n* = 55, HD) and Takeda's (*n* = 111, UPN), the data were analyzed for the same short list of driver genes (Table S2), that is, genes whose alterations are regularly found in AML and considered as driver genes of the disease.

Results are reported in Figure 1 and Table S3. Although few cases (not included), which did not present any mutations in the driver genes studied, remained unclassified (potential class 4), the 166 cases fell into three major classes, listed from the less (class 1) to the most (class 3) severe: *NPM1*‐mutated cases (class 1/Takeda's group B, *n* = 31), cases mutated in genes involved in RNA splicing and epigenetic regulation (class 2/Takeda's, *n* = 66) and *TP53*‐mutated cases (class 3/Takeda's group A, *n* = 69). Mutations in RNA splicing and epigenetic regulation genes define the class of secondary AML [11] or, as recently designated the class of AML with myelodysplasia‐related gene mutations [3, 12]. Actually, class 3 AELs may be secondary too, including to treatment for a previous disease [13].

**FIGURE 1:**
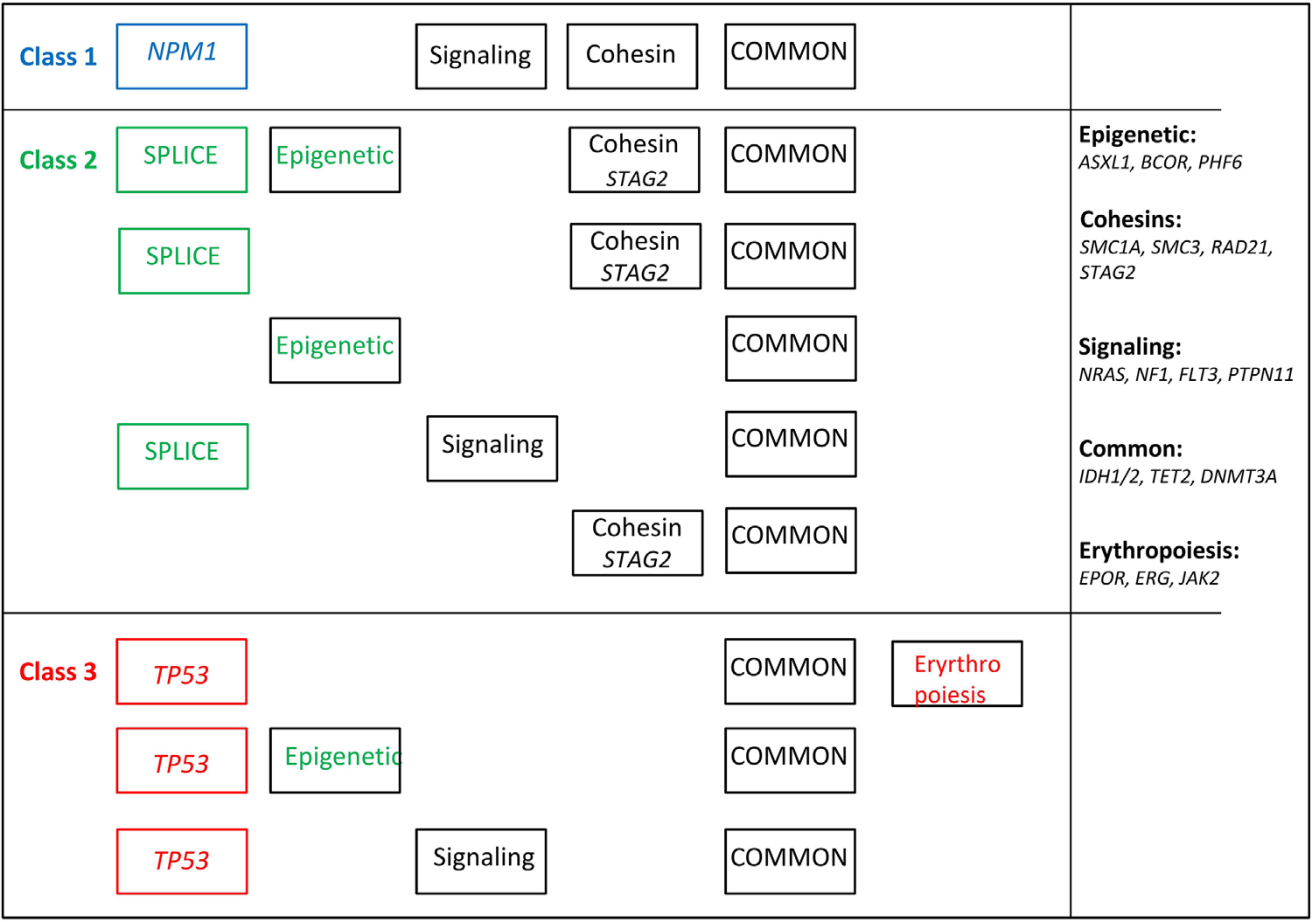
Schematic representation of the combination of the main driver mutations occurring in acute erythroid leukemia (AEL). AEL cases are classified into three major classes according to alterations in driver genes (listed at the right and in Table S2) grouped according to their function.

Some features of this classification can be noticed (Table S3).

1. In classes 1 and 3, mutations of epigenetic, splice, and transcription factors are absent or rare.
2. Mutations in cohesin genes seem differently affected in the three classes: class 1 harbors mutations in *SMC3* (our series) or *SMC1A* (Takeda's), class 2 in *STAG2* (as in [ref. 9]); in class 3, cohesin mutations are absent or rare.
3. Mutations in *TET2, IDH1/2* and *DNMT3A* are found in the three major classes (hence designated “common”).
4. As in the other AMLs, additional alterations can be seen, especially in the*TP53*‐mutated class 3. These are partial or complete losses of chromosome arms 5q, 7q, 17p, and 20q, deletions or breaks of *ETV6* at 12p, and complex karyotypes (not shown).
5. It has been previously reported that pure AELs harbor more than one *TP53* alteration [10, 14, 15]. This applies to nonpure AEL as well. About half of the class 3 cases of our series showed two *TP53* alterations.
6. In class 2, prevalence of cases in men (45/66) could be explained by the fact that many of the driver genes (*STAG2, PHF6, BCOR*) are located on the X chromosome, meaning that only one loss or mutation is enough to completely inactivate the function of the protein, but the prevalence is also observed in class 3 (50/69), and this remains unexplained.
7. Interestingly, mutations in epigenetic and signaling genes (*n* = 66 cases) seem more or less mutually exclusive (only four doubles).

As shown here and in previous reports [5, 6] AEL classification resembles that of non‐AEL AML, with major classes such as *TP53*‐mutated, myelodysplasia‐related and *de novo*‐like *NPM1*‐mutated. Some features are however different, such as the low frequency of *FLT3* alterations and more *TP53* double‐mutations [14, 15].

Alterations in genes involved in erythropoiesis, such as *EPOR* mutations and gains (on chromosome 19), *ERG* gains (on chromosome 21) [8, 10], and *GATA2* mutations may be found, but not in the majority of cases and almost exclusively in AELs with *TP53* mutations (Figure 1) in which they occur in more than half of the cases [8, 10]. The EPOR/JAK2 signaling pathway, when molecularly altered, is functionally active [10] and may be targeted, providing a spark of hope in the AEL therapeutic challenge. However, outside a relatively small group with alterations in signaling pathways (i.e., EPOR/JAK2) and transcriptional regulators (i.e., ERG, GATA2) involved in erythropoiesis, the reason for the particular phenotype of the majority of AELs remains obscure.

Classes 2 and 3 are associated with European LeukemiaNet adverse risk (not shown). The three classes have different prognosis, with class 1 the best and class 3 the worst. Although the difference is not significant, possibly because of the low number of cases, class 2 cases with epigenetic mutations tend to have poorer outcome than class 2 cases with signaling mutations (Figure 2). Distinguishing cases with mutations in either epigenetic or signaling factor (i.e., subclasses) may in the future have an impact on the choice of treatment (e.g., epidrugs vs. kinase inhibitors).

**FIGURE 2:**
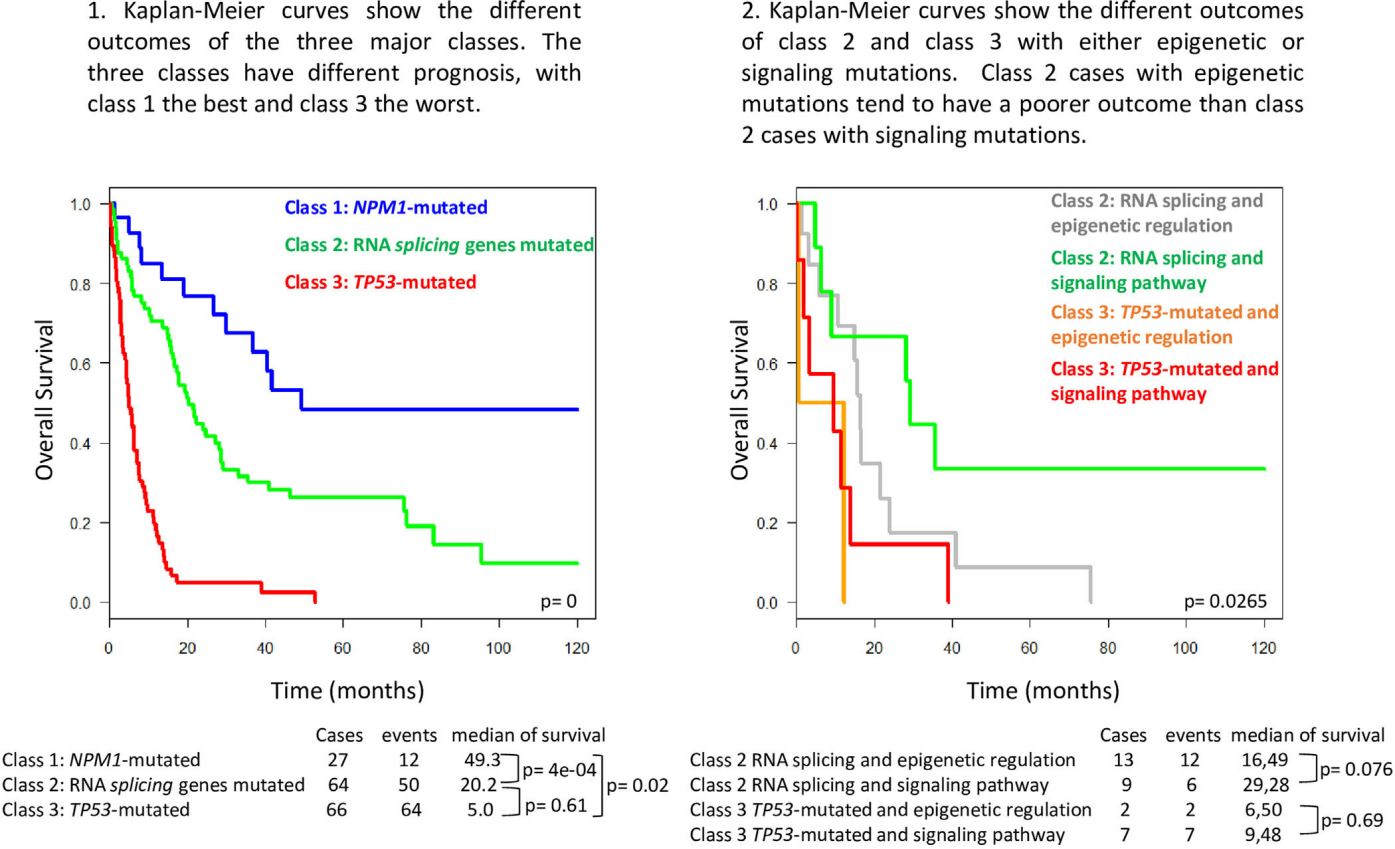
Overall survival of the acute erythroid leukemia (AEL) cohort according to molecular classification. (1) Kaplan–Meier curves show the different outcomes of the three major classes. The three classes have different prognosis, with class 1 the best and class 3 the worst. (2) Kaplan–Meier curves show the different outcomes of class 2 and class 3 with either epigenetic or signaling. Class 2 cases with epigenetic mutations tend to have a poorer outcome than class 2 cases with signaling mutations.

## AUTHOR CONTRIBUTIONS

NC and JA did the analyses and AG the bioinformatics. MH, SG, and NV provided samples and clinical data. DB and VGB designed the study and wrote the paper.

## CONFLICT OF INTEREST STATEMENT

The authors declare that they have no conflict of interest.

## Supporting information

Supporting Information

Supporting Information

Supporting Information

## Data Availability

The data that support the findings of this study are available on request from the corresponding author. The data are not publicly available due to privacy or ethical restrictions.
