# Supplementary material for: Erythroleukemia: Classification
Source: EJHaem. 2023 Mar 17;4(2):450–3. doi: 10.1002/jha2.676 (PMC10188454; doi:10.1002/jha2.676)
Supplement: Supplementary file 2 — Supporting Information [file JHA2-4-450-s002.docx]

**Supplementary Table 2 : List of genes used for the classification**

*NPM1*

*TP53*

Splicing factors : *SF3B1, SRSF2, U2AF1*

Epigenetic regulators : *ASXL1, BCOR, PHF6*

Signaling : *NRAS, NF1* mut/del*, PTPN11, FLT3*

Common (DNA methylation) : *TET2, IDH1, IDH2, DNMT3A*

Transcription factors : *RUNX, CEBPA*

Cohesins : *SMC1A, SMC3, STAG2* mut/del, *RAD21*
